# State-Level Disparities in Residency Applications After *Dobbs v Jackson Women’s Health Organization*

**DOI:** 10.1001/jamanetworkopen.2026.0286

**Published:** 2026-03-02

**Authors:** Anisha P. Ganguly, Anirban Basu, Anna M. Morenz

**Affiliations:** 1 Division of General Medicine and Clinical Epidemiology, Department of Medicine, School of Medicine, University of North Carolina at Chapel Hill, Chapel Hill; 2 Lineberger Comprehensive Cancer Center, University of North Carolina at Chapel Hill, Chapel Hill; 3 The Comparative Health Outcomes, Policy and Economics (CHOICE) Institute, University of Washington School of Pharmacy, Seattle; 4 Division of General Internal Medicine, Department of Medicine, University of Arizona, Tucson; 5 Cancer Prevention and Control Program, University of Arizona Cancer Center, Tucson

## Abstract

**Importance:**

Preliminary analyses have suggested that state-level abortion policy following *Dobbs v Jackson Women’s Health Organization* (*Dobbs*) may affect the health care workforce, including residency applications.

**Objectives:**

To assess disparities in the residency application rate to programs in abortion-restricted states compared with programs in nonrestricted states following the *Dobbs* decision by gender of applicants across all medical specialties and to compare how these differences may vary by specialty.

**Design, Setting, and Participants:**

This cross-sectional study with interrupted time-series analysis used anonymized program-level residency application data from the Association of American Medical Colleges Electronic Residency Application Service (ERAS) from 2019 to 2023. Residency programs across all medical specialties with complete application data across the study period were included. The application cycle immediately prior to the *Dobbs* decision, 2020-2021, was the base year for analysis.

**Exposures:**

New or intensified state-level abortion restrictions enacted post-*Dobbs* in June until October 2022, the 2023 ERAS submission deadline.

**Main Outcomes and Measures:**

The primary outcome was the disparity in application rates to programs between restricted states and nonrestricted states. The application rate was calculated as the number of applications to each program per 100 000 total applications across all programs nationwide annually. Regression models explored the interaction between the exposure and application cycles, controlling for program fixed effects, program size, applicant degree type, and in-state applicants.

**Results:**

Among 4315 residency programs with 22 447 175 total applications (46.1% from women and 53.9% from men), the disparity in the application rate to programs in abortion-restricted states changed by −0.38 (95% CI, −0.70 to −0.05) among women and −0.56 (95% CI, −0.95 to −0.17) among men applying to residency, reflecting an absolute difference of −7833.2 (95% CI, −14 429.7 to −1030.7) applications from women and −12 789.2 (95% CI, −21 695.9 to −3882.4) applications from men to programs in abortion-restricted states. Differences were greater among abortion-related specialties, including obstetrics and gynecology, family medicine, internal medicine, and emergency medicine (−1.17 [95% CI, −1.89 to −0.45] for women and −1.54 [95% CI, −2.39 to −0.69] for men), and decreased among the most competitive specialties (ie, dermatology; neurosurgery; orthopedics; ear, nose, and throat; and plastic surgery) (−0.02 [95% CI, −0.37 to 0.40] for women and −0.13 [95% CI, −0.44 to 0.18] for men).

**Conclusions and Relevance:**

In this cross-sectional panel analysis of residency applications to 4315 programs, the *Dobbs* decision was associated with a significant decrease in residency applications in abortion-restricted states compared with nonrestricted states among both women and men. These findings provide early evidence that *Dobbs* may be associated with state-level disparities in the overall health care training pipeline and workforce.

## Key Points

**Question:**

Did applications from women and men to residency programs in abortion-restricted states decrease compared with programs in nonrestricted states after the *Dobbs v Jackson Women’s Health Organization* Supreme Court decision?

**Findings:**

In this cross-sectional study of residency application data to 4315 residency programs, including 22 447 175 complete applications, across all medical and surgical specialties, applications from both women and men decreased to programs in abortion-restricted states following the *Dobbs* decision.

**Meaning:**

Findings from this study suggest that the *Dobbs* decision was associated with disparities in residency applications from both women and men to programs in abortion-restricted states compared with nonrestricted states, providing evidence that *Dobbs* may contribute to state-level disparities in the overall health care training pipeline and workforce.

## Teaser

This cross-sectional study using an interrupted time-series analysis investigates disparities in the residency application rate to programs in abortion-restricted states compared with nonrestricted states by applicant gender across all medical specialties following the *Dobbs v Jackson Women’s Health Organization* Supreme Court decision.

## Introduction

In June 2022, the US Supreme Court decision of *Dobbs v. Jackson Women’s Health Organization* (hereafter *Dobbs*) overturned decades of precedent set by *Roe v Wade* affirming women’s constitutional right to an abortion.^1^ The *Dobbs* decision deferred abortion policy to state governments, resulting in rapid changes in abortion access that varied from state to state.^2^ In the first year of the ruling, 14 states enacted total bans on abortion, and 7 enacted new restrictions through gestational limits.^3^

Consequences of these state abortion restrictions have been multifaceted and far-reaching. Despite decreased access to and increased travel time for abortion care amid facility closures,^4^ abortion volume rose in 2023 and 2024, in part due to increases in self-management of abortions and telehealth provision of abortion care.^5,6^ Demand for contraception increased substantially, including emergency contraception and procedures for permanent contraception.^7^ Concerningly, infant mortality rates increased, including among neonates with congenital anomalies.^8,9^

In addition to health outcomes, *Dobbs* may affect the health care workforce in abortion-restricted states. In the general labor market, 10 of 18 states banning abortion post-*Dobbs* experienced a decline in female employment growth compared with the national average from 2022 to 2023.^10^ Initial reports suggest that the health care workforce impacts may have a particularly salient association with the specialty of obstetrics and gynecology (OBGYN).^11,12,13,14^ Qualitative research has demonstrated marked moral distress, infringement on professional autonomy, and fear among OBGYN clinicians practicing in states with abortion bans.^11,12^ Zhu et al^13^ noted a 4.2% decrease in the ratio of OBGYN practitioners to women of reproductive age in states with abortion restrictions post-*Dobbs*. These changes also likely influence the health care training pipeline. Hammoud et al^14^ found a small decrease in OBGYN residency applications in abortion-restricted states from 2022 to 2023. A subsequent report from the Association of American Medical Colleges (AAMC) demonstrated decreased residency application rates in abortion-restricted states across all specialties.^15^ Gaps in residency applications between abortion-restricted and nonrestricted states may have long-term consequences for the health care workforce, recognizing that 57.1% of individuals go on to practice in the state of their residency training.^16^

Current research exploring the consequences of the *Dobbs* decision on medical training has largely been limited to descriptive methodologies. Causal inference methods are needed to evaluate the association of abortion restrictions with residency program applications, not only in OBGYN but also in other medical specialties, given that abortion laws may influence trainee decision-making for both professional and personal reasons. The objective of this study was to assess whether disparities in residency application rates per program between abortion-restricted and nonrestricted states changed following the *Dobbs* decision. Disparities in application rates per program were stratified by self-reported gender of applicants. We hypothesized that abortion policy changes would be associated with decreased application rates to programs in restricted states, with a greater decrease among women, and that these differences would be stronger in abortion-related specialties and weaker in the most competitive specialties.

## Methods

In this cross-sectional study with interrupted time-series (ITS) analysis, we analyzed residency program application data from the AAMC Electronic Residency Application Service (ERAS) aggregated at the level of residency programs. Residency programs were anonymized. This study was exempted from review by the University of Washington institutional review board due to the use of deidentified residency program data not meeting criteria for human participants research and followed the Strengthening the Reporting of Observational Studies in Epidemiology (STROBE) reporting guideline for cross-sectional studies.

### Data Source and Study Sample

ERAS and GME Track data were obtained via a custom data request to the AAMC. GME Track is a database of graduate medical education data for residency programs and administrators. The dataset included ERAS residency program application data from the 2018-2019 application cycle to the 2022-2023 application cycle. Applications were submitted during September and October in anticipation of residency program matriculation in June and July of the following year. Data were aggregated by unique program identification number and included medical specialty, number of total applicants to the program, number of applicants by self-reported gender identity (women, men, other, and unknown), number of in-state applicants, number of applicants by degree type (US Doctor of Medicine [MD], Doctor of Osteopathic Medicine [DO], and International Medical Graduate [IMG]), and residency program size. Because there may be a single residency program in a state for smaller specialties, programs were assigned a state-level, abortion policy–related exposure at the time of data request at the AAMC to prevent re-identification of residency programs. Thus, the specific geographic state of programs was not disclosed. Residency programs were limited to those with all 5 years of ERAS data; programs newly accredited or closed during the study period were excluded.

### Exposure Definition

**Figure 1.**
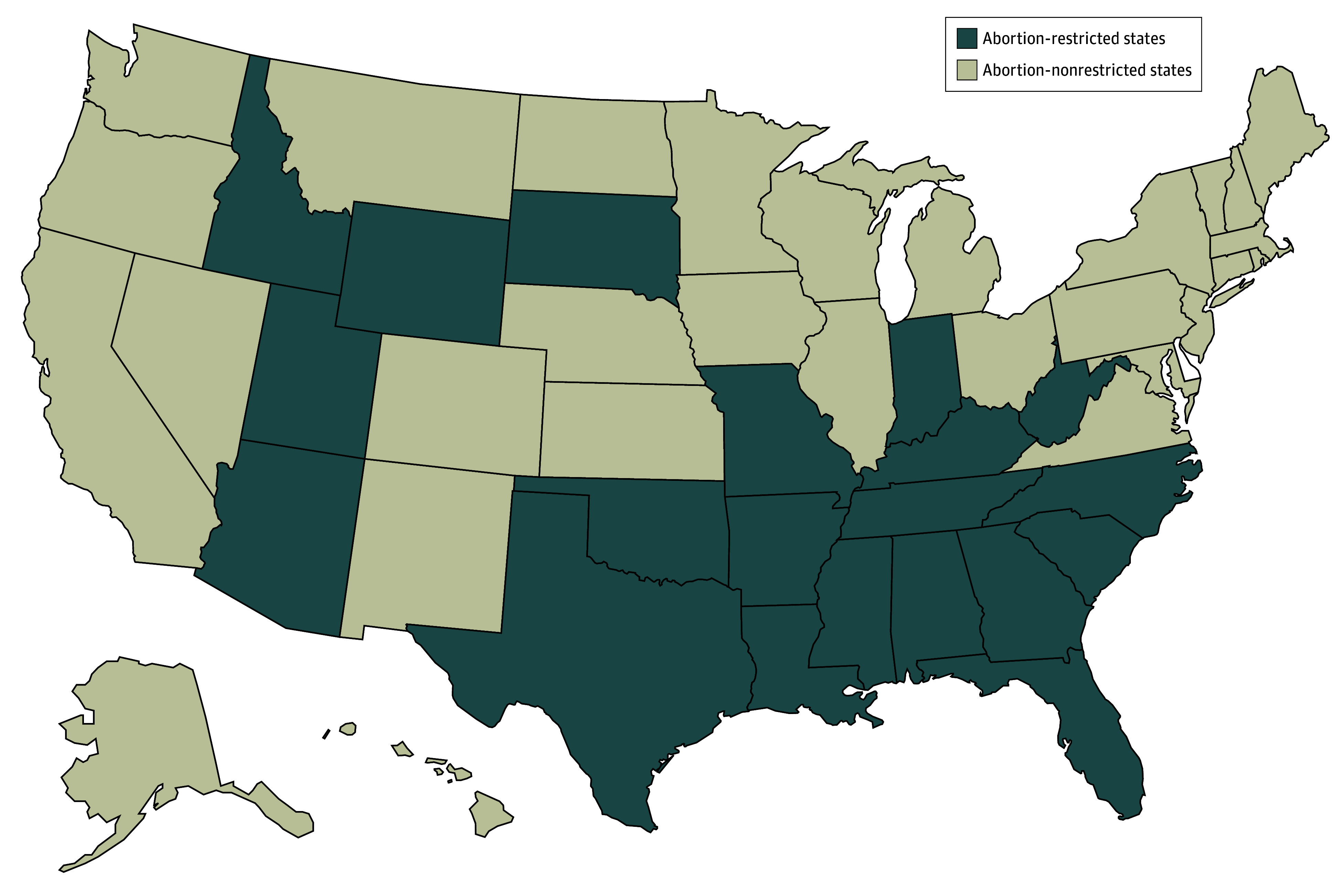
Map of Abortion-Restricted and Nonrestricted States by the October 2022 Intervention Time Point Abortion restriction was defined as any new state abortion restrictions or intensification of existing laws following the *Dobbs v Jackson Women’s Health Organization* decision up to the October 2022 residency application cycle.

The state-level exposure of abortion restriction was a non–time-varying binary variable defined by the presence of any new abortion restrictions introduced or intensified following the *Dobbs* decision in June 2022 up until October 2022, the application submission deadline for the 2022-2023 ERAS season. This time point was selected to designate exposures based on state policies that would be known to applicants up until the time of application submission for the ERAS application cycle following the ruling. State laws enacted following the application deadline were not included in this analysis to prevent exposure misclassification. State laws were coded based on reporting from the KFF Abortion Dashboard and state law tracker.^2,17^ A map of states, their assigned exposure categories, and notes regarding state-level policies in place as of October 2022 are provided in Figure 1.

### Outcomes

The primary outcome was the disparity in application rates to programs between abortion-restricted and nonrestricted states. The application rate was calculated as the number of applications to each program per 100 000 total applications across all programs nationwide annually. The application rate was stratified by self-reported gender. Gender-stratified outcomes were limited to applications from women and men due to insufficient sample size for other gender identities.

### Statistical Analysis

We first summarized the baseline characteristics of residency program application data in 2019 and 2023, stratified by exposure to state abortion restrictions. Then, using an ITS analysis, we estimated the annual trend in the disparity in program application rates between abortion-restricted and nonrestricted states pre- vs post-*Dobbs*. Pre-*Dobbs* included 2018-2019 to 2020-2021 and post-*Dobbs* 2022-2023 ERAS data. Pre-*Dobbs* parallel trends were verified by data visualization. Our empirical specification was identical to running a discrete-time difference-in-differences (DID) model, with the 2021-2022 cycle treated as the base year, with application rates regressed against indicators of chronological years, exposure group, and their interaction, in addition to controlling for program fixed effects, log-transformed program size, proportion of applications by degree type (MD and DO), and proportion of in-state applications. Degree type was included as a covariate to account for longstanding differences in the number of applications submitted by US MD and DO and IMG applicants.^18^ Only the proportions of applications by MD and DO applicants were included due to collinearity with proportion of applications by IMG applicants. Standard errors were clustered at the program level. Since we expected the policy intervention to affect all states, the nonrestricted states could not serve as controls for restricted states. Hence, we interpreted the DID coefficients as a disparity in application rates between exposure groups and followed its trend in an ITS setting. We tested for a stable time-series slope in the disparity outcome during the pre-*Dobbs* period by examining whether the DID coefficients were significantly different from 0 in each of the preexposure years, as well as jointly. To calculate the total shift in applications across exposure groups, we used the following equation: (DID Coefficient/100 000 Total Applications) × (Nonrestricted Programs/Total Programs) × Restricted Programs × Total Applications.

We explored how the changes in the disparity in application rates associated with the *Dobbs* decision may vary by different groups of medical specialties. Abortion-related specialties included OBGYN, family medicine, internal medicine, and emergency medicine, all specialties that may diagnose pregnancy and provide abortion care. Recognizing that applicants to the most competitive specialties may not exercise the same degree of choice of state when applying, we also defined a group of competitive specialties based on the top 5 specialties with the highest rate of unmatched applicants reported by the National Resident Matching Program: dermatology; neurosurgery; orthopedics; ear, nose, and throat; and plastic surgery.^19^ In stratified analyses, we conducted the ITS analysis among (1) abortion-related specialties and nonrelated specialties and (2) competitive and less competitive specialties. We also completed a subgroup analysis of the ITS solely among OBGYN applicants as a validation measure of previous findings demonstrating decreases in applications to abortion-restricted states following *Dobbs*.^14,20,21^

All analyses were conducted using Stata 18, (StataCorp LLC). Two-sided *P* < .05 values were considered statistically significant.

## Results

### Baseline Characteristics and Unadjusted Trends

We examined residency application data from 4315 residency programs that had complete application data from the 2018-2019 season to the 2022-2023 season, including 22 447 175 total applications (46.1% from women and 53.9% from men); 914 programs were excluded due to incomplete data during the study period. Among 4315 programs included, 1417 (32.8%) were in abortion-restricted states and 2898 (67.2%) were in nonrestricted states; among 914 excluded programs, 358 (39.2%) were in abortion-restricted states and 556 (60.8%) were in nonrestricted states. The distribution of residency programs by medical specialty was similar among abortion-restricted and nonrestricted states (eTable in Supplement 1).

In the 2018-2019 application season, programs received a mean (SD) of 971.6 (1002.9) applications for a mean (SD) of 25.8 (23.0) residency trainees (Table 1). The mean (SD) number of applications per program was 431.9 (456.0) from women and 529.9 (572.3) applications from men. The mean (SD) application rate per 100 000 applications nationwide (ie, application rate) from 2018-2019 was 23.2 (23.9). The mean (SD) percentage of in-state applications was 10.7% (10.5%), 42.9% (27.1%) of applications were from US MDs, 14.4% (11.1%) were from US DOs, and 42.7% (27.4%) were from IMGs. In total, 43.9% of the applications were from women, and 56.1% were from men. Baseline differences from 2018-2019 between abortion-restricted and nonrestricted states included a lower percentage of in-state applications (8.2% vs 12.0%), lower percentage of applications from US MDs (41.7% vs 43.4%), and higher percentage of applications from IMGs (44.5% vs 41.8%).

**Table 1.** Residency Program Application Characteristics in 4315 Residency Programs in Abortion-Restricted and Nonrestricted States Before (2018-2019) and After (2022-2023) *Dobbs*

Characteristics of residency program applications	Programs, by application cycle, mean (SD)					
	2018-2019			2022-2023		
	All	In abortion-restricted states	In nonrestricted states	All	In abortion-restricted states	In nonrestricted states
Total applications, No.	971.6 (1002.9)	950.0 (939.9)	982.1 (1032.2)	1060.1 (1111.3)	997.4 (943.4)	1090.7 (1183.7)
Program size, No. of residents	25.8 (23.0)	24.7 (20.8)	26.4 (24.0)	27.3 (23.3)	26.7 (21.5)	27.6 (24.1)
Percentage of in-state applications	10.7 (10.5)	8.2 (7.3)	12.0 (11.6)	10.2 (9.0)	8.7 (7.9)	10.9 (9.5)
Applications from URM applicants, %^a^	22.6 (5.0)	23.6 (5.8)	22.0 (4.5)	28.5 (5.3)	29.5 (5.8)	28.2 (4.9)
Applications from US MDs, %	42.9 (27.1)	41.7 (26.3)	43.5 (27.4)	43.0 (26.5)	42.3 (26.2)	43.4 (26.7)
Applications from US DOs, %	14.4 (11.1)	13.8 (10.1)	14.7 (11.6)	17.6 (11.9)	18.3 (11.6)	17.3 (12.0)
Applications from IMGs, %	42.7 (27.4)	44.5 (27.1)	41.8 (27.5)	39.3 (25.5)	39.4 (25.4)	39.3 (25.6)
Applications from women, %	43.9 (15.7)	43.2 (16.0)	44.2 (15.5)	47.4 (16.0)	46.8 (16.0)	47.8 (16.0)
Applications from men, %	56.1 (15.7)	56.8 (16.0)	55.8 (15.5)	52.3 (16.1)	53.1 (16.0)	52.0 (16.1)
Application rate from women per program^b^	23.2 (24.2)	22.2 (22.4)	23.6 (25.1)	23.2 (23.8)	21.3 (19.9)	24.1 (25.5)
Application rate from men per program^b^	23.2 (24.8)	23.2 (24.8)	23.3 (25.4)	23.2 (26.0)	22.2 (22.7)	23.6 (27.5)

Abbreviations: DO, Doctor of Osteopathic Medicine; *Dobbs*, *Dobbs v Jackson Women’s Health Organization;* IMG, International Medical Graduate; MD, Doctor of Medicine; URM, underrepresented in medicine.

^a^ URM, as defined by the Association of American Medical Colleges, included individuals self-identifying as American Indian or Alaska Native, Black or African American, Hispanic or Latino, or Native Hawaiian or Pacific Islander.

^b^ Application rate defined as number of applications from women and men per program per 100 000 annual applications from women and men nationwide.

**Figure 2.**
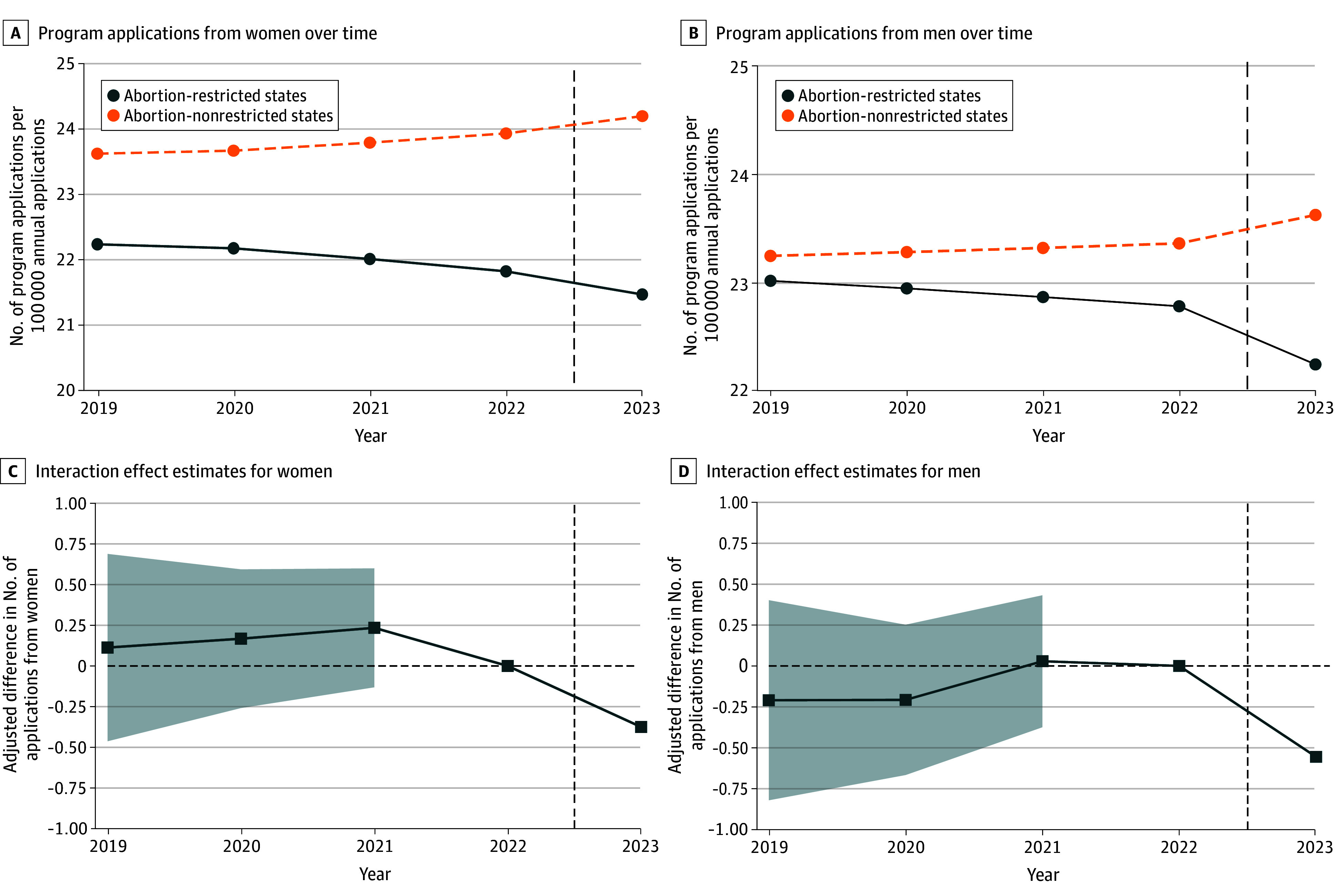
Line Graphs of Unadjusted Trends and Adjusted Percentage Differences in Application Rates per Program From Women and Men in Abortion-Restricted and Nonrestricted States The vertical dashed line in all plots indicates no change in disparity; shading in (C) and (D), 95% CI.

Prior to the *Dobbs* decision, the mean application rate to programs in states with abortion restrictions was lower than to nonrestricted states for women and similar for men (Figure 2). This disparity in application rates from women between abortion-restricted and nonrestricted states widened after *Dobbs*, and a new disparity appeared for men. In the 2021-2022 season, the disparity in application rate from women nationwide was −2.12 (95% CI, −3.57 to −0.68), while for men it was −0.58 (95% CI, −1.00 to 2.16). In an unadjusted regression model, this disparity increased by another −0.60 (95% CI, −0.95 to −0.26) for women and by −0.80 (95% CI, −1.21 to −0.39) for men in the 2022-2023 application cycle post-*Dobbs*. Application rates for all applicants are provided in eFigure 1 in Supplement 1.

### ITS Analysis Before and After *Dobbs*

In the adjusted analysis (Table 2), the disparity in application rates for women changed by −0.38 (95% CI, −0.70 to −0.05) in the 2022-2023 application cycle post-*Dobbs.* Among 2 166 061 applications from women nationwide in the 2022-2023 application cycle, the absolute difference in applications to programs in abortion-restricted compared with programs in nonrestricted states was −7833.2 (95% CI, −14 429.7 to −1030.7).

**Table 2.** Adjusted Regression Analysis of Programs in Abortion-Restricted and Nonrestricted States and Application Rate per Program From Women and Men

	Change in application rate^a^	
	From women, per program (95% CI)	From men, per program (95% CI)
Application cycle		
2018-2019	−1.16 (−1.46 to −0.854)	−1.03 (−1.36 to −0.69)
2019-2020	−0.62 (−0.85 to −0.38)	−0.47 (−0.73 to −0.22)
2020-2021	−0.81 (−1.02 to −0.60)	−0.68 (−0.90 to −0.45)
2021-2022	[Reference]	[Reference]
2022-2023	0.05 (−0.14 to 0.25)	0.12 (−0.09 to 0.34)
Change in disparity of application rate per program in abortion-restricted vs nonrestricted states		
2018-2019	0.11 (−0.47 to 0.69)	−0.21 (−0.82 to 0.40)
2019-2020	0.17 (−0.26 to 0.60)	−0.21 (−0.67 to 0.25)
2020-2021	0.24 (−0.13 to 0.60)	0.03 (−0.38 to 0.43)
2021-2022	[Reference]	[Reference]
2022-2023	−0.38 (−0.70 to −0.05)	−0.56 (−0.95 to −0.17)
Log-transformed program size	3.75 (2.77 to 4.72)	3.36 (2.37 to 4.35)
Proportion of applications from US MDs	−9.01 (−11.0 to −6.99)	−5.71 (−8.09 to −3.32)
Proportion of applications from US DOs	−44.24 (−48.65 to −39.83)	−42.94 (−47.48 to −38.40)
Percentage of in-state applications		
<5	[Reference]	[Reference]
5-12	−2.01 (−2.49 to −1.52)	−2.40 (−2.92 to −1.88)
>12	−4.65 (−5.45 to −3.85)	−5.35 (−6.20 to −4.50)

Abbreviations: DO, Doctor of Osteopathic Medicine; MD, Doctor of Medicine.

^a^ Application rate defined as number of applications from women and men per program per 100 000 annual applications from women and men nationwide.

The change in the disparity in application rates for men in the 2022-2023 application cycle post-*Dobbs* was even wider (−0.56, [95% CI, −0.95 to −0.17]), representing an absolute difference of −12 789.2 (95% CI, −21 695.9 to −3882.4) applications to programs in abortion-restricted states compared with those in nonrestricted states among a total of 2 399 757 applications from men nationwide. For both women and men, no significant changes in disparities were observed in the pre-*Dobbs* years (eg, 0.24 [95% CI, −0.13 to 0.60] for women and 0.03 [95% CI, −0.38 to 0.43] for men in 2021-2021).

Post-*Dobbs* changes in disparities for women (−0.38 [95% CI, −0.70 to −0.05]) and men (−0.56 [95% CI, −0.95 to −0.17]) were not statistically different (*P* = .48). As anticipated, program size (log-transformed size, 3.75 [95% CI, 2.77-4.72] for women and 3.36 [95% CI, 2.37-4.35] for men), applications by degree type (eg, from US MDs, 3.36 [95% CI, 2.37-4.35] for women and −5.71 [95% CI, −8.09 to −3.32] for men), and percentage of in-state applications (eg for >12%, −4.65 [95% CI, −5.45 to −3.85] for women and −5.35 [95% CI, −6.20 to −4.50] for men) were also significantly associated with differences in number of applications per 100 000 applications nationwide for both genders.

### Subgroup and Stratified Analyses

Our subgroup analysis was limited to 846 318 applications to 262 OBGYN residency programs, of which 718 160 (84.9%) were from women and 126 889 (15.0%) were from men (eFigure 2 in Supplement 1). Findings for applications from women were consistent with the primary analysis; there was a significant change in the disparity in application rates for women by −0.66 (95% CI, −1.30 to −0.02) after *Dobbs*. However, in contrast to findings in the overall sample, there was no significant change for men (−0.04 [95% CI, −0.20 to 0.19]).

In stratified analyses (Table 3), the disparity in application rates was greater for abortion-related specialties (−1.17 [95% CI, −1.89 to −0.45] for women and −1.54 [95% CI, −2.39 to −0.69] for men) post-*Dobbs*, whereas there were no significant changes in abortion-unrelated specialties. In the stratified analysis of most competitive specialties, there were no significant changes in the disparities in application rates for either for women or men. Among less competitive specialties, findings were consistent with the primary ITS analysis (−0.43 [95% CI, −0.81 to −0.06] for women, −0.61 [95% CI, −1.06 to −0.16] for men).

**Table 3.** Specialty-Stratified Analyses of Change in Disparity of Application Rate per 100 000 Applications for Programs in Abortion-Restricted vs Nonrestricted States, by Gender

Application cycle	Change in disparity of application rate per 100 000 applications (95% CI)							
	Abortion-related specialty		Abortion-unrelated specialty		Most competitive specialties^a^		Less competitive specialties	
	Women	Men	Women	Men	Women	Men	Women	Men
2018-2019	0.26 (−1.06 to 1.57)	−0.28 (−1.67 to 1.10)	0.02 (−0.34 to 0.37)	−0.16 (−0.55 to 0.23)	0.08 (−0.24 to 0.39)	0.05 (−0.33 to 0.42)	0.07 (−0.60 to 0.73)	−0.32 (−1.03 to 0.38)
2019-2020	0.32 (−0.65 to 1.28)	−0.15 (−1.17 to 0.86)	0.08 (−0.17 to 0.33)	−0.23 (−0.53 to 0.07)	−0.04 (−0.30 to 0.21)	−0.34 (−0.70 to 0.02)	0.19 (−0.31 to 0.68)	−0.22 (−0.75 to 0.31)
2020-2021	0.001 (−0.82 to 0.82)	−0.19 (−1.07 to 0.69)	0.31 (0.08 to 0.53)	0.08 (−0.18 to 0.34)	−0.08 (−0.37 to 0.21)	−0.13 (−0.56 to 0.31)	0.25 (−0.18 to 0.67)	0.003 (−0.46 to 0.47)
2021-2022	[Reference]	[Reference]	[Reference]	[Reference]	[Reference]	[Reference]	[Reference]	[Reference]
2022-2023	−1.17 (−1.89 to −0.45)	−1.54 (−2.39 to −0.69)	0.13 (−0.08 to 0.34)	0.07 (−0.21 to 0.34)	0.02 (−0.37 to 0.40)	−0.13 (−0.44 to 0.18)	−0.43 (−0.81 to −0.06)	−0.61 (−1.06 to −0.16)

^a^ The top 5 most competitive specialties included dermatology; neurosurgery; orthopedics; ear, nose, and throat; and plastic surgery.

## Discussion

In this cross-sectional study with an ITS analysis of 22 447 175 applications to 4315 residency programs for 5 years across all medical specialties, we observed a statistically significant disparity in applications from both women and men in states with abortion restrictions following the *Dobbs* decision in 2022 compared with states that did not enact restrictions. Despite overall increases in the number of residency applications during the study period, existing disparities between application volume to programs in abortion-restricted and nonrestricted states widened for women applying to residency, and new disparities emerged for men applying to residency post-*Dobbs*. Stratified analyses suggested that specialty type may influence differences, as effect sizes were increased among abortion-related specialties and decreased among the most competitive specialties.

These findings affirm and expand on recent studies demonstrating decreased OBGYN residency applications and applicant interest in abortion-restricted states following the *Dobbs* decision.^19^ Additional studies have reported challenges faced by OBGYN programs in abortion-restricted states, including nonadherence to accreditation standards requiring abortion training, financial constraints for medical training, and burnout among residents and program leadership.^12,21,22^ This study upholds this previous research and further quantifies the differences in applications between programs in abortion-restricted and nonrestricted states following the *Dobbs* decision using causal inference methods.

This study advances research on the health care workforce impacts of the *Dobbs* decision by examining the association of state abortion restrictions with residency applications to all specialties beyond the discipline of OBGYN. Although OBGYNs have historically delivered the majority of abortions,^23,24,25^ most abortions in the US are managed as medication abortions rather than procedures requiring surgical training.^26^ The specialties of family medicine, internal medicine, and emergency medicine increasingly consider medication abortion within their scope of practice and support training in abortion care.^25,27,28^ In stratified analyses, we found that disparities in application rate in abortion-restricted states following the *Dobbs* decision were magnified in these abortion-related specialties, suggesting that training changes may extend beyond OBGYN to family medicine, internal medicine, and emergency medicine residency programs. Declining interest in family medicine, internal medicine, and emergency medicine residency programs in abortion-restricted states may exacerbate existing shortages in primary care and disparate access to emergency care.^29,30^ Previous studies have forecasted shortages in the OBGYN workforce, affecting not only abortion care but also regular obstetric and gynecologic care^13,31^; our study results suggest that there may be similar consequences to the primary care and emergency medicine workforce in abortion-restricted states.

Another novel finding in this study was segmentation of disparities in application rates among abortion-restricted and nonrestricted states post-*Dobbs* by gender. Prior to the *Dobbs* decision, there were preexisting differences in applications from women among programs in abortion-restricted and nonrestricted states that further widened after 2022. Preexisting disparities among applications from women may reflect both professional and personal factors that intensified following *Dobbs.* Previous research has implicated overall reproductive health climate and access to family planning services in labor market representation of women.^32^ Even before *Dobbs*, state legislatures worked to reduce access to abortion care; in 2021 alone, 108 state laws were enacted to restrict access to telemedicine for medication abortion, introduce new gestational limits, and restrict coverage for abortion.^33^ State regulation of abortion also closely correlates with regulation of contraception and family planning services,^34,35^ which may explain the gap in residency applications among women that existed in abortion-restricted states prior to the *Dobbs* decision and further increased after the ruling.

We found a new decrease in application volume from men in 2023 following the *Dobbs* decision. This finding may indicate a more global change associated with the *Dobbs* decision for all residency applicants, regardless of gender, compared with piecemeal state-level changes for women prior to the ruling. Professional factors, such as physician autonomy, moral distress, and fear of legal prosecution,^12,36,37^ affect both women and men in their training and practice. Personal factors driving applicant behavior post-*Dobbs* may include not only abortion care but also family planning decisions and contraception access.^22^ These factors apply to both women as well as men, who may choose nonrestricted states out of consideration of their own family planning and reproductive health care access for their partners and children.^22^ Moreover, the majority of men in 2024 supported legal access to abortion,^38^ and men with higher levels of education, as with medical trainees, are more likely to support access to abortion.^39^ These findings were not demonstrated in the subgroup analysis of men applying to OBGYN residencies in the present study, which may be due to limited sample size. Among 177 133 applicants to OBGYN residencies in 2023, 22 399 (12.6%) were men. Recent studies have discussed decreased training opportunities and patient preferences for women OBGYNs as external drivers of a longstanding trend in decreasing applications from men to OBGYN residencies.^40^ Nonetheless, our primary study findings highlight the early association of these state policies with decreases in residency applications from both women and men and overall threats to the health care workforce, regardless of gender, in abortion-restricted states.

### Limitations

Our study has important limitations. Exposure assignment was based on state policies enacted as of fall 2022 following the Supreme Court ruling and did not include enjoined legislation (laws that were passed but halted due to pending court action); however, enjoined legislation may reflect a negative reproductive climate that could influence applicant behavior. For example, 2 states, Iowa and North Dakota, had enjoined abortion restrictions at the intervention time point that resulted in new abortion laws enacted in July 2024 and April 2023, respectively.^2^ Classifying these states as unexposed in this analysis could have biased findings toward the null, as applicants could have been aware of the possibility of new abortion restrictions pending court action. Data were aggregated at the program level; thus, demographic information at the applicant level was not available. Demographic information was presented as percentages of applications received by programs. The dataset was solely composed of ERAS data without match data from the National Resident Matching Program; thus, the ultimate gender composition of matched residents is not presented. There are no data reflecting applicant quality or competitiveness in this analysis. Importantly, states with abortion restrictions also commonly introduced other legislation, such as anti–diversity, equity, and inclusion and anti–gender-affirming care measures, that may similarly negatively impact applicant interest.^41,42^ Additional factors that may drive applications, such as cost of living, resident salary, and unionization, were not adjusted for in this analysis. In addition, state policies following the *Dobbs* decision have evolved rapidly, with states introducing both new abortion restrictions and abortion protections following our intervention time point of October 2022. As of this writing, 2 states (Arizona and Missouri) that were classified as abortion-restricted at our intervention time point introduced state constitutional amendments to protect abortion rights in the 2024 election, and an abortion ban in 1 restricted state (Wyoming) has been enjoined since March 2023.^2,43^ Three states that were nonrestricted in October 2022 (Iowa, Nebraska, and North Dakota) have introduced abortion restrictions since that time point.^2^ Nonetheless, the findings of this study, conducted up to the application cycle immediately following the *Dobbs* decision, provide the earliest and likely most conservative estimates of changes associated with *Dobbs* among residency applications. These rapidly changing state policies necessitate ongoing analyses with contemporaneous data to provide the most current assessments of impacts to the health care workforce.

## Conclusions

This cross-sectional study with ITS analysis found that the *Dobbs* decision was associated with significant decreases in residency applications from both women and men in abortion-restricted states compared with nonrestricted states. These differences were more pronounced for specialties more likely to provide or refer for abortion care, including primary care. Widening state-level disparities in residency applications have implications for residency programs, the health care workforce, and long-term access to health care beyond reproductive care in the post-*Dobbs* era.

## Notes

Supplement 1. **eTable.** Distribution of Residency Programs by Medical Specialty in Abortion-Restricted and Nonrestricted States **eFigure 1.** Unadjusted Trends and Adjusted % Difference in Application Rate per Program From All Applicants in Abortion-Restricted and Nonrestricted States **eFigure 2.** Unadjusted Trends and Adjusted % Difference in Application Rate per Program from Women and Men to OBGYN Programs in Abortion-Restricted and Nonrestricted States

Supplement 2.
Data Sharing Statement
