# Errors in Methods, Results, and Article Information

**DOI:** 10.1001/jamanetworkopen.2026.6890

**Published:** 2026-03-26

In the Original Investigation titled “State-Level Disparities in Residency Applications After *Dobbs v Jackson Women’s Health Organization*,”^1^ published on March 2, 2026, there were errors in the data sources listed in the Methods and the total number of applications reported for the overall and subgroup analyses in the Abstract Results, Key Points Findings, and main Results. In addition, a disclaimer was missing from the Article Information. This article was corrected online.^1^
