# Supplementary material for: State-Level Disparities in Residency Applications After Dobbs v Jackson Women’s Health Organization
Source: JAMA Netw Open. 2026 Mar 2;9(3):e260286. doi: 10.1001/jamanetworkopen.2026.0286 (PMC12954540; doi:10.1001/jamanetworkopen.2026.0286)
Supplement: Supplement 1. — eTable. Distribution of Residency Programs by Medical Specialty in Abortion-Restricted and Nonrestricted States eFigure 1. Unadjusted Trends and Adjusted % Difference in Application Rate per Program From All Applicants in Abortion-Restricted and Nonrestricted States eFigure 2. Unadjusted Trends and Adjusted % Difference in Application Rate per Program from Women and Men to OBGYN Programs in Abortion-Restricted and Nonrestricted States [file jamanetwopen-e260286-s001.pdf]

Supplementary Online Content

Ganguly AP, Basu A, Morenz AM. State-level disparities in residency applications after *Dobbs v Jackson Women's Health Organization*. *JAMA Netw Open*. 2026;9(3):e260286. doi:10.1001/jamanetworkopen.2026.0286

eTable. Distribution of Residency Programs by Medical Specialty in Abortion-Restricted and Non-Restricted States

eFigure 1. Unadjusted Trends and Adjusted % Difference in Application Rate per Program From All Applicants in Abortion-Restricted and Non-Restricted States

eFigure 2. Unadjusted Trends and Adjusted % Difference in Application Rate per Program From Women and Men to OBGYN Programs in Abortion-Restricted and Non-Restricted States

This supplementary material has been provided by the authors to give readers additional information about their work.

© 2026 Ganguly AP et al. *JAMA Network Open*.

eTable. Distribution of Residency Programs by Medical Specialty in Abortion-Restricted and Non-Restricted States

Characteristics of residency programs, mean (SD)	All Programs	Programs in Abortion-Restricted States	Programs in Non-Restricted States
**Number of programs**	4,315 (100.0%)	1,417 (32.8%)	2,898 (67.2%)
**Medical specialty**			
Anesthesia	142 (3.3%)	45 (3.2%)	97 (3.4%)
Dermatology	123 (2.9%)	43 (3.1%)	80 (2.8%)
Emergency medicine	229 (5.3%)	74 (5.3%)	155 (5.4%)
Family medicine	596 (13.9%)	202 (14.3%)	394 (13.7%)
Internal medicine	497 (11.6%)	157 (11.2%)	340 (11.8%)
Neurosurgery	108 (2.5%)	36 (2.6%)	72 (2.5%)
Neurology	225 (5.2%)	80 (5.7%)	145 (5.0%)
OBGYN	262 (6.1%)	83 (5.9%)	179 (6.2%)
Orthopedic surgery	158 (3.7%)	54 (3.8%)	104 (3.6%)
ENT	108 (2.5%)	36 (2.6%)	72 (2.5%)
Pathology	135 (3.1%)	46 (3.3%)	89 (3.1%)
Pediatrics	197 (4.6%)	70 (5.0%)	127 (4.4%)
Physical Medicine and Rehabilitation	87 (2.0%)	24 (1.7%)	63 (2.2%)
Plastic Surgery	63 (1.5%)	18 (1.3%)	45 (1.6%)
Psychiatry	246 (5.7%)	82 (5.8%)	164 (5.7%)
Radiation oncology	82 (1.9%)	25 (1.8%)	57 (2.0%)
Radiology	271 (6.3%)	79 (5.6%)	192 (6.7%)
Surgery	436 (10.2%)	141 (10.0%)	295 (10.2%)
Urology	117 (2.7%)	43 (3.1%)	74 (2.6%)
Combined internal medicine programs	168 (3.9%)	58 (4.1%)	110 (3.8%)
Combined pediatrics programs	37 (0.9%)	12 (0.9%)	25 (0.9%)

© 2026 Ganguly AP et al. *JAMA Network Open*.

eFigure 1. Unadjusted Trends and Adjusted % Difference in Application Rate per Program From All Applicants in Abortion-Restricted and Non-Restricted States

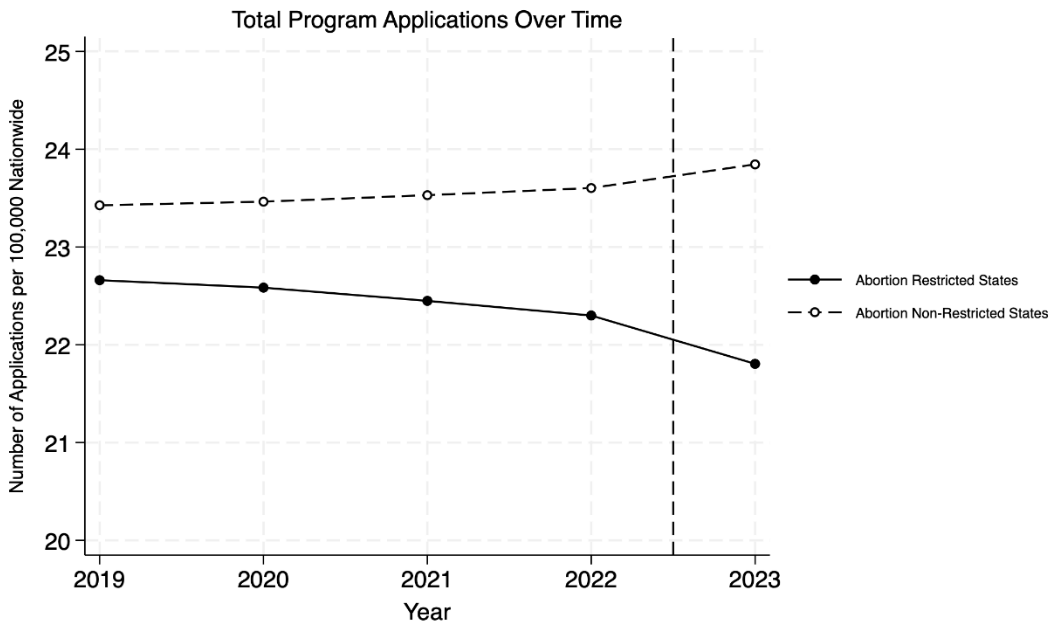

a.

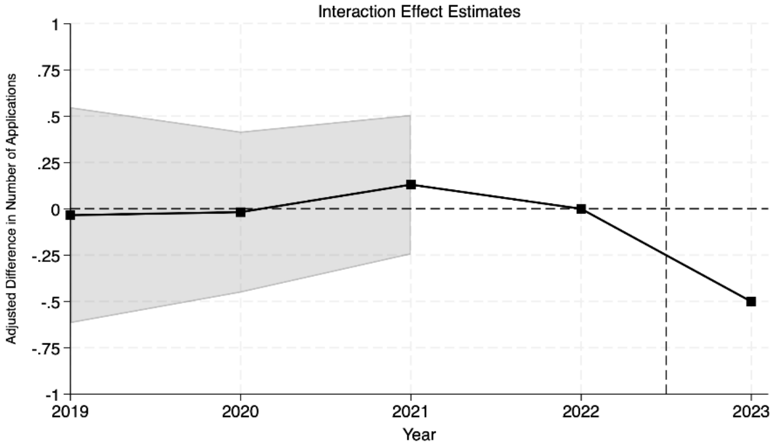

b.

© 2026 Ganguly AP et al. *JAMA Network Open*.

eFigure 2. Unadjusted Trends and Adjusted % Difference in Application Rate per Program From Women and Men to OBGYN Programs in Abortion-Restricted and Non-Restricted States

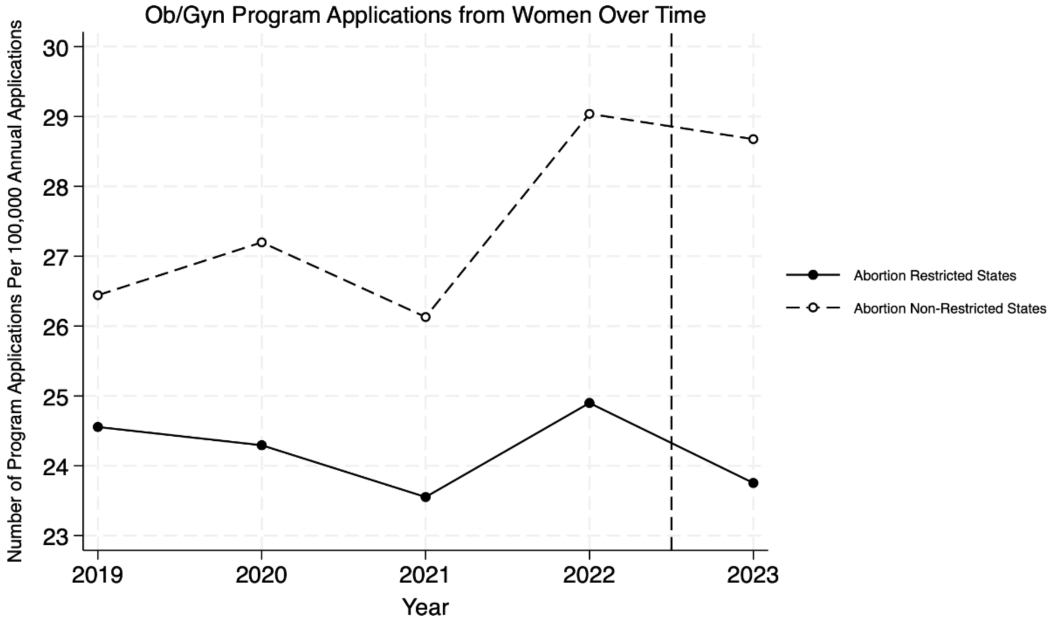

a.

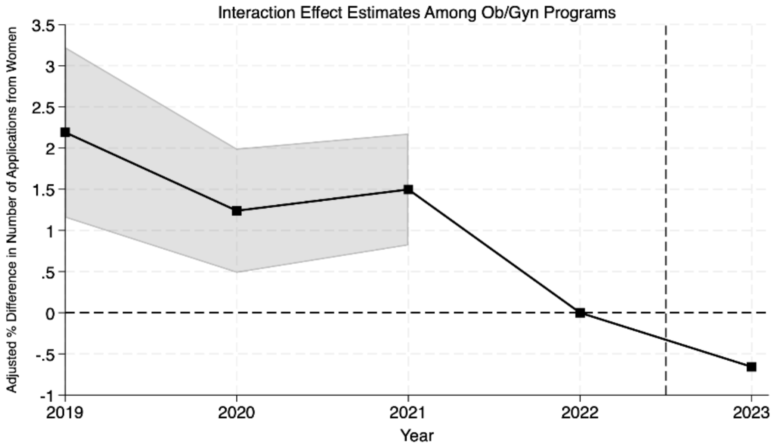

b.

© 2026 Ganguly AP et al. *JAMA Network Open*.

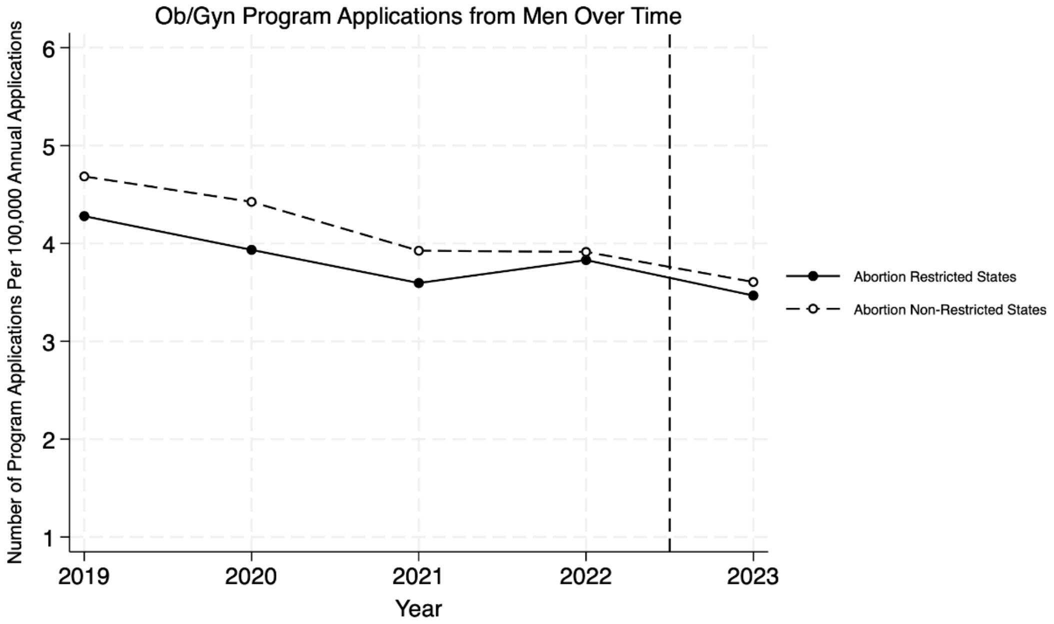

c.

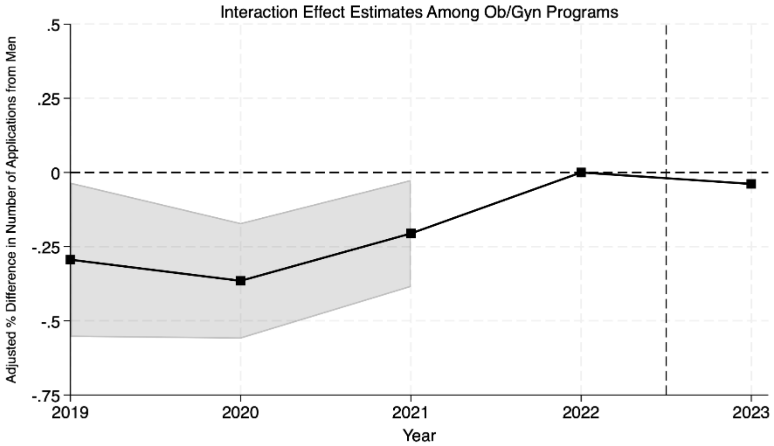

d.

© 2026 Ganguly AP et al. *JAMA Network Open*.
