# Supplementary material for: State-Level Disparities in Residency Applications After Dobbs v Jackson Women’s Health Organization
Source: JAMA Netw Open. 2026 Mar 2;9(3):e260286. doi: 10.1001/jamanetworkopen.2026.0286 (PMC12954540; doi:10.1001/jamanetworkopen.2026.0286)
Supplement: Supplement 2. — Data Sharing Statement [file jamanetwopen-e260286-s002.pdf]

Data Sharing Statement

Ganguly. State-Level Disparities in Residency Applications After Dobbs v Jackson Women's Health Organization. *JAMA Netw Open*. Published March 02, 2026. doi:10.1001/jamanetworkopen.2026.0286

Data
Data available: Yes
Data types: Other (please specify)
Additional Information: These data were made available by a custom data request to the Association of American Medical Colleges (AAMC). The dataset was shared with the authors under a data licensing agreement with their institutions. Data sharing of this dataset is contingent on a data licensing agreement with the AAMC and the study authors.
How to access data: These data were made available by a custom data request to the Association of American Medical Colleges (AAMC). The dataset was shared with the authors under a data licensing agreement with their institutions. Data sharing of this dataset is contingent on a data licensing agreement with the AAMC and the study authors.
When available: With publication

Supporting Documents
Document types: Statistical/analytic code
How to access documents: Contact corresponding author.
When available: With publication

Additional Information
Who can access the data: Anyone requesting the data.
Types of analyses: For any purpose.
Mechanisms of data availability: Data sharing of this dataset is contingent on a data licensing agreement with the AAMC and the study authors.
